# Effect of Cementitious Materials on the Engineering Properties of Lightweight Aggregate Mortars Containing Recycled Water

**DOI:** 10.3390/ma15051967

**Published:** 2022-03-07

**Authors:** Jae-In Lee, Sung-Ho Bae, Ji-Hwan Kim, Se-Jin Choi

**Affiliations:** Department of Architectural Engineering, Wonkwang University, Iksan 54538, Korea; csj2378@hanmail.net (J.-I.L.); csj2378@naver.com (S.-H.B.); csj2378@gmail.com (J.-H.K.)

**Keywords:** recycled water, blast furnace slag powder, fly ash, strength, ternary cementitious mortar, carbonation depth

## Abstract

With the trend toward taller and larger structures, the demand for high-strength and lightweight cement concrete has increased in the construction industry. Equipment for transporting ready-mixed concrete is frequently used to bring concrete to construction sites, and washing this equipment generates a large amount of recycled water, which is an industrial by-product. In this study, we recycled this water as the pre-wetting water for lightweight aggregate and as mixing water, and we substituted blast furnace slag powder (BS) and fly ash (FA) as cementitious materials (Cm). In addition, we evaluated the fluidity, compressive strength, tensile strength, drying shrinkage, and accelerated carbonation depth of lightweight ternary cementitious mortars (TCMs) containing artificial lightweight aggregate and recycled water. The 28-day compressive strengths of the lightweight TCM specimens with BS and FA were ~47.2–51.7 MPa, except for the specimen with 20% each of BS and FA (40.2 MPa), which was higher than that of the control specimen with 100% OPC (45.9 MPa). Meanwhile, the 28-day tensile strengths of the lightweight TCM specimens containing BS and FA were ~2.81–3.20 MPa, which are ~13.7–29.5% higher than those of the control specimen. In this study, the TCM specimen with 5% each of BS and FA performed the best in terms of the combination of compressive strength, tensile strength, and carbonation resistance.

## 1. Introduction

Cement concrete, which is widely used in various fields in the construction industry, must demonstrate improved performance, such as high strength and lightweight, with the increasing demand for taller and larger structures. Ready-mixed concrete, which is mainly used at construction sites, must be transported to these sites, and the equipment used to that end undergoes a washing process after the concrete is poured. This process generates a large amount of recycled water, an industrial by-product. Some of this water is recovered through recycling facilities, but some companies use recycled water that exceeds the standard value or illegally discharge this water into rivers, causing environmental pollution [1]. To solve these environmental problems, several studies have attempted to recycle the by-products of ready-mixed concrete, such as recycled water [2,3,4,5,6,7,8,9].

Xuan et al. [2] reported that applying the slurry to concrete effectively reduces its drying shrinkage after the accelerated carbonation of slurry waste generated in a ready-mixed concrete plant. Zervaki et al. [5] examined the characteristics of mortar mixed with dry sludge and sludge water generated in a ready-mixed concrete plant and reported that using sludge water increased its compressive strength. Sandrolini et al. [8] evaluated the characteristics of mortar and concrete mixed with ready-mixed concrete waste wash water, reporting that this water improved their durability.

Moreover, worldwide efforts to suppress global warming are required, and the cement and concrete industry emits a large amount of greenhouse gases [10,11]. In the construction industry, cement substitutes, such as blast furnace slag powder (BS) or fly ash (FA), are widely used to reduce the amount of cement used as a part of efforts to reduce greenhouse gases or to improve the durability of concrete [12,13]. In particular, several studies have recently attempted to increase the number of cement substitutes by mixing ternary cementitious material (Cm) with two or more cement substitutes [14,15,16,17,18,19,20,21]. Upon mixing metakaolin and silica fume into the mortar, Chu et al. [16] found that although silica fume negatively affected workability, metakaolin could alleviate this negative effect. Similarly, Andrade et al. [18] investigated the properties of ternary cementitious paste with metakaolin and nanosilica and demonstrated that adding 15% metakaolin and 3% nanosilica increased the compressive strength by ~44% compared to that of the control specimen without these two additives. Meanwhile, after evaluating the durability of ternary concrete with BS, FA, and limestone filler, Lauch et al. [21] reported that BS and FA improved the chloride penetration resistance of concrete.

Although many studies have used the by-products of the ready-mixed concrete industry and Cm, no research has yet been published on ternary cementitious composites with artificial lightweight aggregates and recycled water. In this study, we used recycled water as both the pre-wetting water for the lightweight aggregate and the mixing water, and we substituted BS and FA as Cm. In addition, we evaluated the fluidity, compressive strength, tensile strength, drying shrinkage, and accelerated carbonation depth of lightweight ternary cementitious mortars (TCMs) containing artificial lightweight aggregate and recycled water.

## 2. Materials and Methods

### 2.1. Materials

The Cm used in this study was ASTM type-I OPC manufactured by the Asia Cement Co. (Seoul, Korea), and the BS was obtained from Daehan Slag Co., Ltd., Gwangyang-si, Korea. FA manufactured at the D thermal power plant in Korea was used.

**Figure 1:**
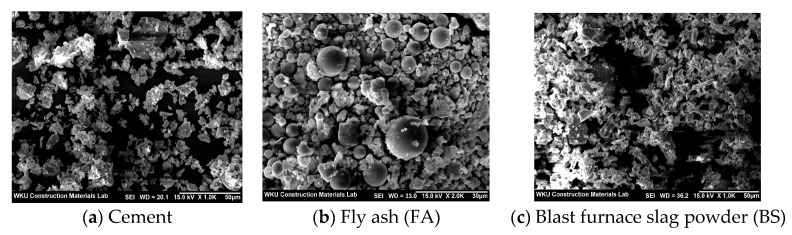
SEM images of cement (**a**), FA (**b**), and BS (**c**).

Figure 1 shows the SEM images of the cement, FA, and BS used in this study. Unlike FA, which is composed of spherical particles, the cement and BS have irregularly shaped grains. Table 1 lists the chemical compositions of the Cm used in this study.

**Figure 2:**
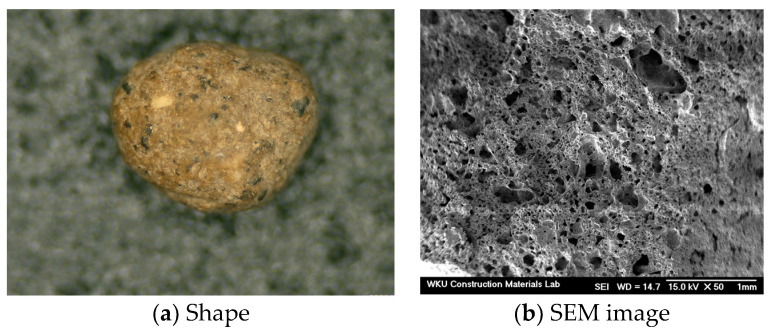
Optical (**a**) and SEM (**b**) images of the artificial lightweight aggregate.

As an artificial lightweight sand (LS), lightweight fine aggregate from KOEN, Korea, manufactured by calcining coal ash and dredged soil at ~1100–1200 °C, was used. The shape of the internal voids in the LS aggregate can affect the flowability and mechanical properties of the mortar sample. The optical micrograph of a single grain of the artificial LS in Figure 2a shows its shape, and the SEM image in Figure 2b reveals the grain interior, which contains a large number of voids. Table 2 lists its physical properties.

In the case of recycled water, by referring to a previous study [22], sludge was prepared with a 4:1 ratio of cement and sand–fines (less than 0.15 mm), and recycled water with a sludge content of 5% was used as the pre-wetting and mixing water. Table 3 details the composition of the sludge used in this study.

### 2.2. Mix Proportions and Specimen Preparation

Table 4 shows the mix proportions of the experimental cement mortar specimens.

The water–Cm ratio was fixed at 45%, and the sludge content of the recycled water was 5%, which showed good characteristics in a previous study [23]. The recycled water was used as both the pre-wetting water and mixing water. To make TCMs, 5–20% BS and FA were used to replace part of the cement content.

Cubic specimens with dimensions of 50 mm × 50 mm × 50 mm were prepared via molding for compressive strength testing, and cylindrical specimens with dimensions of ø50 mm × 100 mm were prepared for split-tensile strength testing. In addition, 40 mm × 40 mm × 160 mm specimens were prepared for drying shrinkage and carbonation tests. We then demolded the specimens after 24 h and cured them in a water tank at 20 °C until the required age (7, 28, or 56 days). Mortar flow and compressive strength were measured according to KS L 5105 [24], and tensile strength was determined according to KS F 2423 [25]. Drying shrinkage was assessed using a contact gauge according to KS F 2424 [26]. For the carbonation test, the carbonation depth was measured using a phenolphthalein solution after a carbonation process in an accelerated carbonation chamber according to KS F 2584 [27].

## 3. Results and Discussion

### 3.1. Mortar Flow

**Figure 3:**
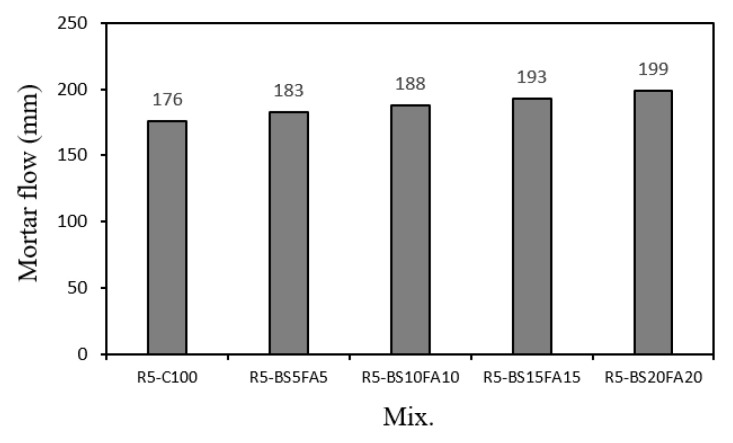
Mortar flow.

Figure 3 presents the different flow values of the lightweight aggregate mortar samples with recycled water and ternary Cm. The flow of the R5-C100 sample was the lowest at ~176 mm. The flow values of the TCM samples with BS and FA were all higher than that of R5-C100. Further, the flow of the mortar sample gradually increased with the amount of FA and BS. This increased flow was attributed to the spherical shape of FA, as shown in Figure 1, which likely caused a ball bearing effect, rather than BS, which was irregularly shaped.

The flow of the R5-BS20FA20 sample, which had the highest amounts of BS and FA, was ~199 mm, which was ~13.3% higher than that of R5-C100. Furthermore, each time the amount of BS and FA was increased by 5%, the flow value of the lightweight TCM sample with recycled water increased by ~2.6–4.2%. It has been reported that the use of recycled water does not have a significant effect on the mortar flow [4]; similarly, this study found that the mortar flow was more affected by the use of Cm than the use of recycled water.

### 3.2. Compressive Strength

**Figure 4:**
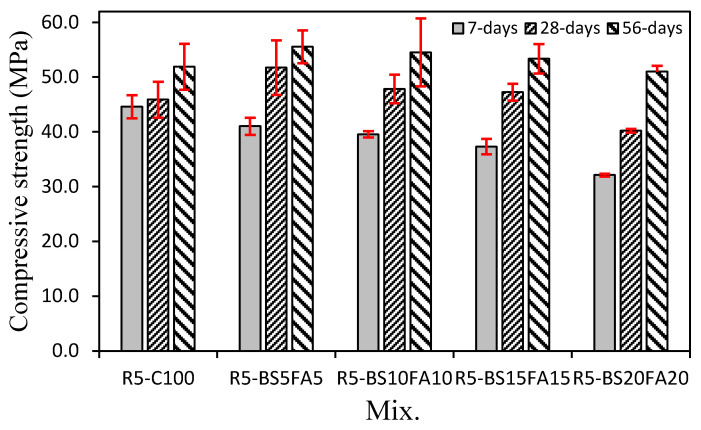
Compressive strength.

Figure 4 shows changes in the compressive strength of the lightweight mortar specimens with recycled water and ternary Cm. After 7 days, the compressive strength of R5-C100 was ~44.6 MPa, showing the highest value. The 7-day compressive strengths of the TCM specimens with BS and FA were ~32.1–41.0 MPa, all of which were lower than that of R5-C100. Moreover, as the amount of BS and FA increased, the 7-day compressive strength of the TCM specimen tended to decrease.

However, the evolution of the 28-day compressive strength showed a different trend. Specifically, the 28-day compressive strength of the R5-C100 control specimen was ~45.9 MPa, whereas the 28-day compressive strengths of the lightweight TCM specimens containing BS and FA were ~47.2–51.7 MPa; only the R5-BS20FA20 specimen showed a lower compressive strength (40.2 MPa) than R5-C100. In particular, R5-BS5FA5 showed the highest 28-day compressive strength of ~51.7 MPa, which was ~12.8% higher than that of R5-C100.

Interestingly, the R5-BS15FA15 specimen also showed a higher 28-day compressive strength of ~47.3 MPa, although its total Cm amount was 30%. This significant enhancement in performance might have been due to the filling action of fines in the recycled water [8] and the activation of the Cm reaction owing to the high alkalinity of the recycled water [28,29,30]. Therefore, these results suggest that the appropriate use of recycled water and Cm in a lightweight aggregate cement composite can effectively improve its compressive strength.

After 28 days, the strength continued to evolve, and the 56-day compressive strength of the R5-C100 specimen was ~51.9 MPa. In contrast, the compressive strength of R5-BS5FA5 (55.5 MPa) was the highest among the TCM specimens. Indeed, the 56-day compressive strengths of most TCM specimens with Cm were higher than that of R5-C100 without Cm; only the 56-day compressive strength of the R5-BS20FA20 specimen (51.0 MPa) was similar to that of the R5-C100 specimen.

### 3.3. Tensile Strength

**Figure 5:**
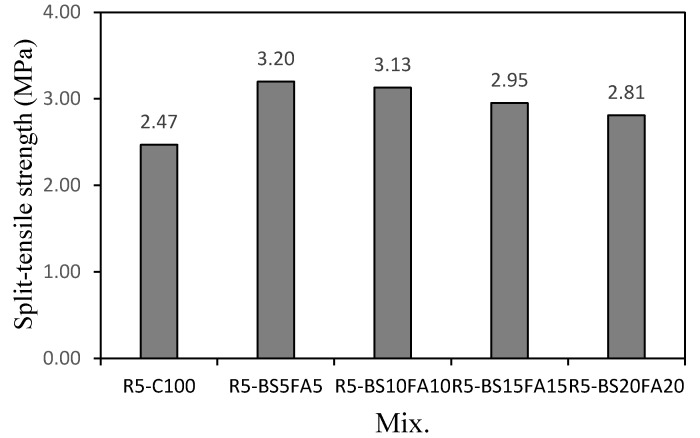
Tensile strength.

Figure 5 compares the 28-day tensile strengths of the lightweight TCM specimens with recycled water and ternary Cm with that of the control. The R5-C100 specimen without Cm showed the lowest tensile strength of ~2.47 MPa, whereas those of the lightweight TCM specimens with BS and FA were ~2.81–3.20 MPa, which were ~13.7–29.5% higher than that of R5-C100. In particular, the tensile strength of the R5-BS5FA5 specimen, which had the highest 28-day compressive strength, was ~3.20 MPa, the highest among the mixtures and ~29.5% higher than that of R5-C100. This increase compared to that of the control is more than double compared to that in the compressive strength (12.8%). Therefore, the proper use of recycled water and Cm in the lightweight aggregate cement composite effectively increases both the tensile and compressive strengths of the cement composites. In this study, the improvement in the tensile strength was greater.

### 3.4. Drying Shrinkage

**Figure 6:**
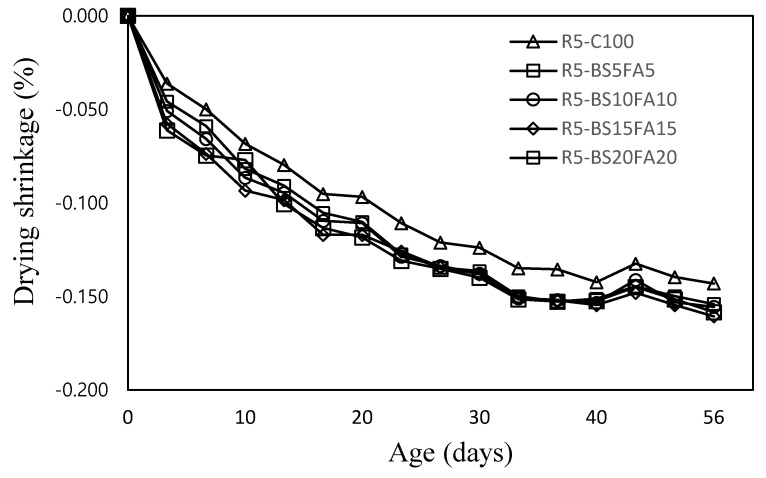
Drying shrinkage.

The drying shrinkage of lightweight mortar specimens with recycled water and ternary Cm is shown in Figure 6. After 56 days, the R5-C100 specimen without Cm showed the lowest drying shrinkage at ~0.143% compared with the lightweight TCM specimens incorporating BS and FA. Specifically, the 56-day drying shrinkage of R5-BS15FA15 was ~0.161%, which was ~12.5% greater than that of R5-C100, whereas that of R5-BS5FA5 (0.154%) was relatively low among the TCM specimens. The higher drying shrinkage of the TCM specimens with Cm compared with that of the R5-C100 specimen without Cm was likely due to the increase in the mineral admixture content and the high fineness effect of the Cm particles used in this study [31,32].

### 3.5. Carbonation Depth

**Figure 7:**
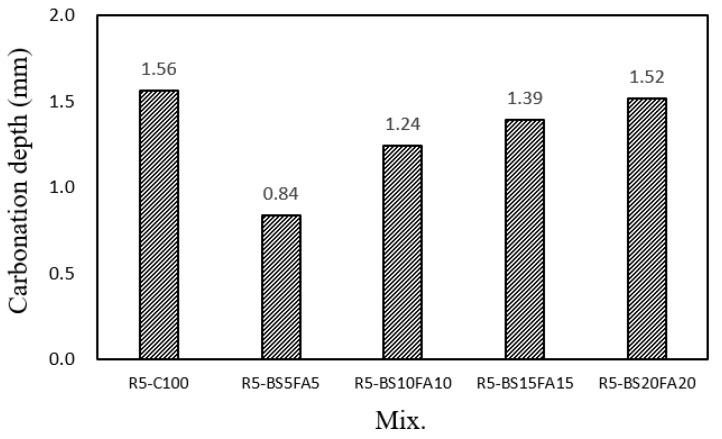
Carbonation depth.

Figure 7 shows the carbonation depths of the lightweight mortar specimens with recycled water and ternary Cm after 28 days of accelerated carbonation. The carbonation depth of the R5-C100 specimen without Cm was ~1.56 mm, whereas those of the lightweight TCM specimens with Cm were ~0.84 to 1.52 mm, all of which were smaller than that of R5-C100. However, as the amount of BS and FA increased, the carbonation depths of the TCM specimens gradually increased. In particular, the carbonation depth of R5-BS5FA5 was only ~0.84 mm, which was ~46.1% smaller than that of R5-C100. The reason for this decreased carbonation depth was that the 56-day compressive strength of the R5-BS5FA5 specimen was the largest, and a denser cement matrix is considered to improve its penetration resistance to CO_2_ gas. Thus, this study found that the proper use of recycled water and Cm improved the compressive strength of the mortar by making its internal structure denser, which seems to have influenced the observed improvement in the carbonation resistance. Therefore, in this study, the R5-BS5FA5 showed the optimal performance in terms of its combination of compressive strength, tensile strength, and carbonation resistance.

## 4. Conclusions

(1) In this study, when the amounts of BS and FA were increased by 5%, the flow value of the lightweight TCM specimen with recycled water increased by ~2.6–4.2%.
(2) The 28-day compressive strengths of the lightweight TCM specimens containing BS and FA were ~47.2–51.7 MPa, except for that of R5-BS20FA20 (40.2 MPa), which was higher than that of R5-C100. In particular, the 28-day compressive strength of the R5-BS5FA5 specimen with 5% BS and FA was ~51.7 MPa, which was ~12.8% higher than that of R5-C100.
(3) The 28-day tensile strengths of the lightweight TCM specimens incorporating BS and FA were ~2.81–3.20 MPa, which were ~13.7–29.5% higher than that of the R5-C100 specimen. Therefore, the proper use of recycled water and Cm in lightweight aggregate cement composites is efficacious in improving the tensile and compressive strengths of the cement composite specimen.
(4) The drying shrinkage of the lightweight TCM specimens with BS and FA was relatively higher than that of the R5-C100 specimen.
(5) The carbonation depth of the R5-BS5FA5 specimen with 5% BS and FA was ~0.84 mm, which was ~46.1% smaller than that of R5-C100. The carbonation depths of all the lightweight TCM specimens containing Cm were ~0.84–1.52 mm and were smaller than that of the R5-C100 specimen (1.56 mm).

In this study, the R5-BS5FA5 specimen incorporated with 5% each of BS and FA showed the best performance in terms of compressive strength, tensile strength, and carbonation resistance.

## Figures and Tables

**Table 1:** Chemical composition of cementitious materials (cm).

Type	SiO_2_	Al_2_O_3_	Fe_2_O_3_	CaO	MgO	K_2_O	Blaine (cm^2^/g)	Density (g/cm^3^)
Cement	17.43	6.50	3.57	64.40	2.55	1.17	3430	3.15
Blast furnace slag powder (BS)	30.61	13.98	0.32	40.71	6.43	0.60	4210	2.93
Fly ash (FA)	64.88	20.56	6.06	2.58	0.80	1.45	3710	2.21

**Table 2:** Physical properties of lightweight fine aggregate.

Type	Fineness Modulus (FM)	Density	Water Absorption Ratio (%)	Unit Weight (kg/L)
Artificial lightweight sand (LS)	4.61	1.77	8.71	1010

**Table 3:** Composition of the sludge.

Mix.	W/C (%)	Water (g)	Cement (g)	Sand Fines (g)
Sludge	50	200	400	100

**Table 4:** Mix proportions of the cement mortar specimens.

Mix.	BS (%)	FA (%)	Sludge Content (%)	LS (S * %)	W/Cm (%)	W (kg/m^3^)	Cm (kg/m^3^)
R5-C100	0	0	5	100	45	153	340
R5-BS5FA5	5	5					
R5-BS10FA10	10	10					
R5-BS15FA15	15	15					
R5-BS20FA20	20	20					

## Data Availability

Not applicable.
